# Co-occurrence patterns and the large-scale spatial structure of benthic communities in seagrass meadows and bare sand

**DOI:** 10.1186/s12898-020-00308-4

**Published:** 2020-07-08

**Authors:** Casper Kraan, Simon F. Thrush, Carsten F. Dormann

**Affiliations:** 1 Helmholtz Institute for Functional Marine Biodiversity At the University of Oldenburg, Ammerländer Heerstraße 231, 23129 Oldenburg, Germany; 2 Department of Functional Ecology, Alfred Wegener Institute Helmholtz Centre for Polar and Marine Research, Am Handelshafen 12, 27570 Bremerhaven, Germany; 3 Institute of Marine Science, University of Auckland, Private Bag 92019, Auckland, 1142 New Zealand; 4 Biometry and Environmental System Analysis, University of Freiburg, Tennenbacherstr. 4, 79106 Freiburg, Germany; 5 Present Address: Thünen Institute of Sea Fisheries, Herwigstraße 31, 27572 Bremerhaven, Germany

**Keywords:** Co-occurrence, Environmental variation, Intertidal, Joint species distribution model, Seagrass meadows

## Abstract

**Background:**

Species distribution models are commonly used tools to describe diversity patterns and support conservation measures. There is a wide range of approaches to developing SDMs, each highlighting different characteristics of both the data and the ecology of the species or assemblages represented by the data. Yet, signals of species co-occurrences in community data are usually ignored, due to the assumption that such structuring roles of species co-occurrences are limited to small spatial scales and require experimental studies to be detected. Here, our aim is to explore associations among marine sandy-bottom sediment inhabitants and test for the structuring effect of seagrass on co-occurrences among these species across a New Zealand intertidal sandflat, using a joint species distribution model (JSDM).

**Results:**

We ran a JSDM on a total of 27 macrobenthic species co-occurring in 300,000 m^2^ of sandflat. These species represented all major taxonomic groups, i.e. polychaetes, bivalves and crustaceans, collected in 400 sampling locations. A number of significant co-occurrences due to shared habitat preferences were present in vegetated areas, where negative and positive correlations were approximately equally common. A few species, among them the gastropods *Cominella glandiformis* and *Notoacmea scapha*, co-occurred randomly with other seagrass benthic inhabitants. Residual correlations were less apparent and mostly positive. In bare sand flats shared habitat preferences resulted in many significant co-occurrences of benthic species. Moreover, many negative and positive residual patterns between benthic species remained after accounting for habitat preferences. Some species occurring in both habitats showed similarities in their correlations, such as the polychaete *Aglaophamus macroura,* which shared habitat preferences with many other benthic species in both habitats, yet no residual correlations remained in either habitat.

**Conclusions:**

Firstly, analyses based on a latent variable approach to joint distributions stressed the structuring role of species co-occurrences beyond experimental scales. Secondly, results showed context dependent interactions, highlighted by species having more interconnected networks in New Zealand bare sediment sandflats than in seagrass meadows. These findings stress the critical importance of natural history to modelling, as well as incorporating ecological reality in SDMs.

## Background

Co-occurrence patterns of species across a landscape may arise due to shared habitat preferences, dispersal patterns, community interactions (e.g. facilitation, competition) or the interaction of these processes [1, 2]. This interest in joint distributions of species relates to (1) incorporating real-world complexity in species distribution models (SDM) by allowing species to interact [3], and (2) solving technical challenges posed by having to extend generalized linear mixed models to multivariate models that allow us to separate correlation patterns across multiple species from environmental responses [1].

Biotic interactions have been experimentally shown to be important drivers of local community structure in many different kinds of ecological systems (e.g. [4]). Their role in driving patterns at a larger scale, even determining species ranges, is less well established, although clear examples exist [3, 5]. At large scales, manipulative experiments are unfeasible, and detection of interactions relies on correlative evidence, recently in the form of joint analyses of species abundances or occurrences (joint species distribution models: JSDMs). With this method, environmental drivers of species distributions are accounted for, and remaining correlation in the model residuals indicate association between species, often interpreted as biotic interactions (e.g. [6, 7]).

**Fig. 1:**
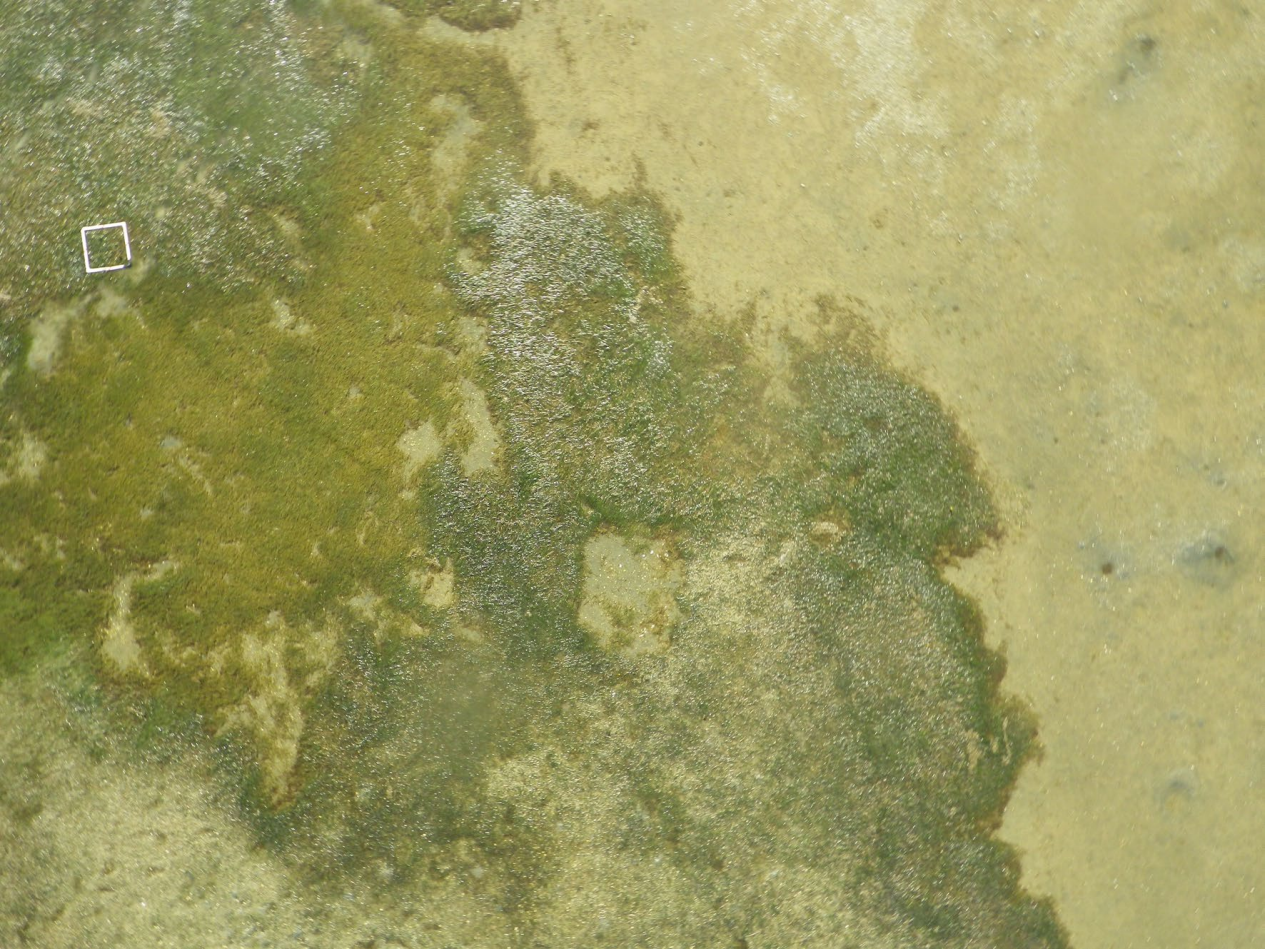
An illustration of the visual contrast between seagrass meadows (left hand side) and bare sediment sandflats (right hand side). Picture taken by Roman Zajac at Kaipara harbour, New Zealand. The white rectangle encompasses 0.5 × 0.5 m

Competitive and facilitative interactions may arguably be stronger among near-sessile organisms, such as plants, parasites, or indeed benthic invertebrates [8]. Structure provided by the habitat may substantially affect the way such interactions play out, providing physical shelter or habitat for predators [9]. Here, our aim is employing macroecological techniques to explore associations among marine sandy-bottom sediment inhabitants and test for the structuring effect of seagrass on co-occurrences among these species. Specifically, we test the hypothesis that macrozoobenthic communities in seagrass patches (*Zostera muelleri*) maintain more interconnected interaction networks across intertidal areas than the same species communities inhabiting intertidal sandflats (Fig. 1). This reflects the common assumption that seagrass meadows, due to their structural complexity, play a significant role in maintaining diversity and resilience of coastal systems [10, 11].

Our main interest lies in the ecological inference possible from multi-species models. We acknowledge the technical challenges and great benefits of employing JSDMs and refer to ([1, 12] and references cited herein) for mathematical comparisons between modelling choices. To address the lack of knowledge regarding the structuring role of species co-occurrences across landscapes, our work employed latent variable models (LVM) and demonstrated their advantages for studying multi-species distributions. LVMs are emerging as efficient tools to dissect multivariate data [1, 13], since they explicitly link latent variables to each sample as unobserved predictors to capture unobserved environmental predictors [1] or non-random co-occurrences. We showed that patterns in species co-occurrences were context dependent and structure communities across spatial scales of at least 1 km, as illustrated by comparing marine species communities in seagrass meadows to the same species occupying bare intertidal sandflats. These results have implications for most current SDM research, which are mainly employing single-species models.

## Results

We ran a joint species distribution analysis on a total of 27 species co-occurring in 300,000 m^2^ of New Zealand intertidal sandflat, representing all major taxonomic groups, i.e. polychaetes, bivalves and crustaceans. Eleven species only occurred in seagrass meadows, 6 species were restricted to bare sand flats, whereas 10 species were common (> 25% of sampling locations) to both habitats (Table 1).

**Table 1:** Species descriptions and information on their occurrences (*n* sampling locations) and abundances (mean count) in seagrass (coverage at least 33%) and bare-sand systems (0% coverage of seagrass)

			Seagrass	Bare sand
		*n*-samples	63	279
Species	Abbreviation	Taxonomic group	Occurrence (mean)	Occurrence (mean)
*Aglaophamus macroura*	aglmac	Polychaetes	16 (0.46)	99 (0.52)
*Aonides trifida*	aontri	Polychaetes	36 (2.87)	161 (12.35)
*Arthritica bifurcata*	artbif	Bivalves	15 (0.60)	
*Austrovenus stutchburyi*	ausstu	Bivalves	33 (3.97)	146 (1.71)
*“Bumpy” cirrisyllid*	bumcir	Polychaetes	15 (0.60)	97 (1.19)
*Colurostylus lemurum*	collem	Crustaceans		176 (1.63)
*Cominella glandiformis*	comgla	Gastropods	22 (0.62)	
*Halicarcinus varius*	halvar	Crustaceans	15 (0.40)	
*Halicarcinus whitei*	halwhi	Crustaceans	15 (0.43)	
*Hesionidea*	hesion	Polychaetes		97 (0.68)
*Heteromastus filiformis*	hetfil	Polychaetes	36 (1.16)	145 (1.44)
*Macomona liliana*	maclil	Bivalves	61 (3.89)	261 (7.00)
*Macroclymenella stewartensis stewardensisstewartensis*	macste	Polychaetes	26 (0.97)	
*Magelona dakini*	magdak	Polychaetes		152 (1.66)
*Nemertean*	nemert	Polychaetes	30 (1.02)	202 (2.19)
*Notoacmea scapha*	notsca	Gastropods	24 (0.71)	
*Nucula hartvigiana*	nucchar	Bivalves	45 (2.03)	95 (1.65)
*Orbinia papillosa*	orbpap	Polychaetes		100 (0.95)
*Owenia petersonae*	owepet	Polychaetes	20 (1.16)	
*Paphies australis*	papaus	Bivalves	19 (0.51)	133 (5.07)
*Paracalliope novizealandiae*	parnou	Crustaceans	30 (5.22)	103 (1.05)
*Phoronis sp.*	phoron	Other		91 (1.17)
*Platynereis australis*	plaaus	Polychaetes	25 (0.59)	
*Prionospio aucklandica*	priauc	Polychaetes	31 (2.37)	
*Pseudopolydora ‘FAT’*	psefat	Polychaetes	27 (1.06)	
*Soletellina siliqua*	solsil	Bivalves		155 (3.56)
*Trochodota dendyi*	troden	Polychaetes	22 (0.46)	

**Fig. 2:**
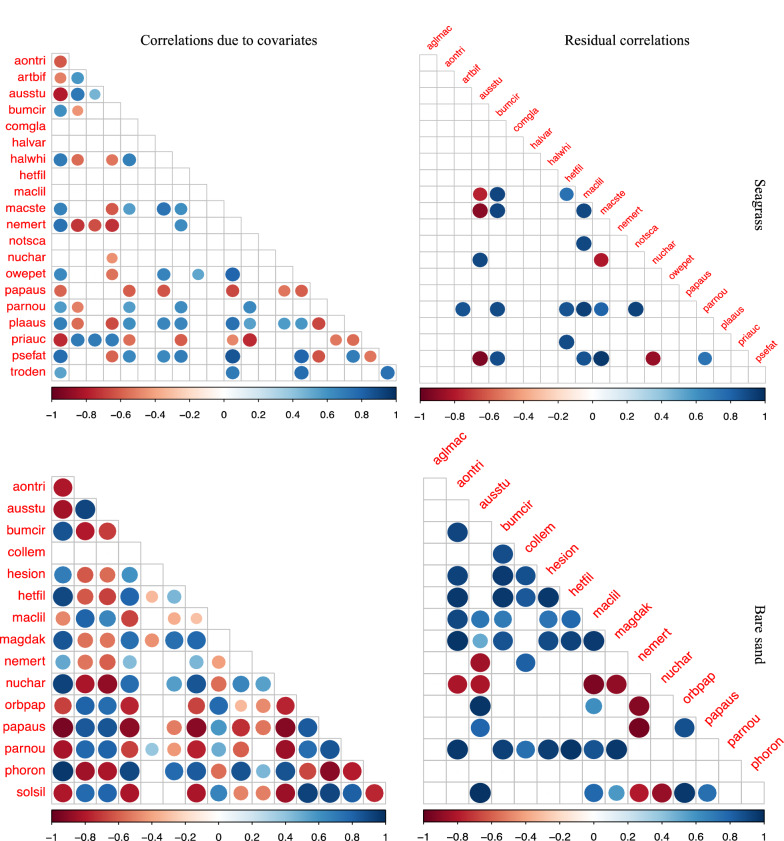
Correlation between species occurring in seagrass meadows (top) or bare sand flats (bottom) due to shared habitat preferences (left panel) or residual correlation (right panel) as modelled by LVM (see Table 1 for full species names). Results are based on the most parsimonious model. Only correlations which differ from 0 are shown, i.e. the larger the bubble size the more different from 0, where red indicates negative values and blue positive correlation

A number of significant co-occurrences due to shared habitat preferences were present in vegetated areas (Fig. 2), where negative and positive correlations were approximately equally common. A few species, among them the gastropods *Cominella glandiformis* and *Notoacmea scapha*, co-occurred randomly with other seagrass benthic inhabitants, as indicated by an empty row of raw correlations (Fig. 2). Residual correlations were less apparent and mostly positive (Fig. 2). *Austrovenus stutchburyi*, a common suspension-feeding bivalve, was the only species to display negative residual correlations with more than a single species, i.e. the deposit-feeding bivalve *Macomona liliana* and two tube-building polychaetes *Macroclymenella stewartensis* and *Pseudopolydora* ‘FAT’.

In bare sand flats shared habitat preferences resulted in many significant co-occurrences of benthic species. Moreover, many negative and positive residual patterns between benthic species remained after accounting for habitat preferences (Fig. 2). Most noteworthy, the deposit-feeding bivalve *Nucula hartvigiana* was negatively correlated with deposit-feeding polychaetes *Aonides trifida* and *Magelona dakini*, the suspension-feeding bivalves *A. stutchburyi* and *Soletellina siliqua*, and the deposit-feeding bivalve *M. liliana*.

Some species occurring in both habitats showed similarities in their correlations, such as the polychaete *Aglaophamus macroura,* which shared habitat preferences with many other benthic species in both habitats, yet no residual correlations remained in either habitat. The amphipod *Paracalliope novizealandiae* not only co-occurred with many other species in both habitats, but also displayed positive residual correlation with six or more species in each habitat (Fig. 2).

## Discussion

Acknowledging the inherent complexity of species distributions requires methods that are capable of accommodating real-world species associations. Here we used LVM to discern the joint distribution of species (see, e.g., [1, 7], showing that across a large intertidal area (300,000 m^2^) both positive and negative shared habitat preferences are abundant. Also, we demonstrated that positive and to some extend negative co-occurrences of species are common, particularly in bare sand flats.

Contrary to our expectations, seagrass meadows mainly structured benthic communities due to species having a shared habitat preference, whereas in bare sandflats species commonly displayed positive and negative co-occurrences in addition to shared habitat preferences. This might indicate that in a more obvious physically structured habitat, such as provided by seagrass meadows [11], provisioning of different niches leads to the observed community structure. Also, the physical structure of seagrass meadows might limit the mobility of some species like *Austrovenus stutchburyi*, as they can't bulldoze their way through the rhizome mat. Most species are also moving (especially in early benthic stages) associated with hydrodynamics and bedload transport and this effect will also be baffled by seagrass. In bare sand flats, which are often perceived as seemingly featureless habitats (but see [14]), community structure likely is more animal created, with greater dominance of interference competition and facilitation to mediate the impact of abiotic stress [15]. Such species interactions are critical in habitat modifications, influencing sediment compositions, hydrodynamics, and biogeochemistry, thereby impacting interaction networks, feedbacks and ecosystem functioning [16]. Lastly, how mobile animals perceive the mosaic of patterning might impact their dispersal through the seascape [17, 18].

As reviewed in detail by Dormann et al*.* [7] and in the introduction, we refrain from discussing our results in the context of biotic interactions. Given that assessing co-occurrences are based on residual correlations (e.g. [13, 19]), unmeasured environmental variables rather than biotic interactions might also explain observed patterns. We considered most commonly used habitat features to describe benthic community patterns (see, e.g., [20, 21]) and we acknowledge that this data-driven approach offered hints on potential community organisation processes [19]. For example, random occurrences in seagrass meadows, as shown by the living attached to seagrass blades limpet *Notoacmea scapha* and the bulldozing predator/scavenging whelk *Cominella glandiformis*, might indicate a life-style utilizing other habitat features than the surrounding benthic fauna that mostly live buried in the sediment. In New Zealand intertidal systems, the suspension-feeding *A. stutchburyi* and the deposit-feeding *Macomona liliana* are key bioturbating bivalves regarding ecosystem architecture due to their sediment-dwelling life-style [16, 20, 22–24]. High densities of adult *M. liliana* negatively affect microphytobenthos and juveniles of many other species including conspecifics due to their grazing behaviour [24], and its negative co-occurrence with tube-building and sediment stabilizing polychaetes *Macroclymenella stewartensis* and *Pseudopolydora* ‘fat’ in seagrass meadows suggests such opposing "interests". The bivalve *Nucula hartvigiana* negatively co-occurred with, e.g., the polychaetes *Aonides trifida* and *Magelona dakini*, in intertidal sandflats. This might indicate subtle differences in habitat preferences, since *N. hartvigiana* tends to be in slightly muddy and organic rich sediments, whereas both polychaetes like more permeable low organic load sediments (e.g. [25, 26]). The amphipod *Paracalliope novizealandiae* flits around at the sediment water interface, co-occurring with many species in both habitats, perhaps profiting from their sediment reworking activities to gather food.

## Conclusions

Positive or negative co-occurrence of species, after considering habitat preferences, are common and are present beyond experimentally accessible spatial scales. This strengthens previous research aiming to resolve biodiversity-ecosystem functioning at seascape-scales, which noted the importance of scale considerations to understand structure and resilience of ecosystems [11, 15, 21, 27]. In addition to underlining the spatial scale of community structure, our work shows the context-dependent nature of species co-occurrences, in which the higher relative importance of positive and negative co-occurrences in bare sand flats, harbouring to a greater extend the same species as neighbouring seagrass meadows, lead to a highly interconnected benthic network. These findings stress the critical importance of natural history to modelling, as well as incorporating ecological reality in SDMs.

From the management point of view, this work has important implications; frequently, bare sand bottoms are undervalued compared to, for example, reefs, which are considered of larger importance for conservation when taking decisions in coastal management (e.g. development of harbours, marinas, construction of pipelines). Nevertheless, our work shows that at least some bare sand bottoms harbour rich benthic communities with complex ecological interactions that are worth conserving.

## Methods

### Empirical data

To test whether the co-occurrence of species influences the structure of communities beyond experimental scales, we used data on macrobenthic fauna, mainly bivalves, polychaetes, and crustaceans, and environmental features from 400 sampling points arrayed across 300,000 m^2^ of intertidal sandflat in Kaipara Harbour, New Zealand [20, 23]. Effectively these sampling points covered the complete intertidal sandflat exposed during low-tide. This enabled us to model spatial co-occurrences from 30 cm (smallest sampling distance) to 1 km (largest sampling distance). Specifically, we compared species distributed across starkly contrasting mosaics of seagrass patches (seagrass coverage at least 33%; 63 sampling points; Additional file 1: Table S1), and bare intertidal sandflats (0% coverage of seagrass; 279 sampling points; Additional file 2: Table S2). In both habitats, separated by 30 cm up to 1 km, we only considered species present in > 25% of the sampling locations (Table 1 for species names and occurrence information), to warrant sufficient power to assess species-to-species co-occurrences and to deal only with species ecologically associated with bare sand or seagrass habitats.

### Model description

Latent variable models are extensions of multivariate regression models with unobserved ("latent") predictors that help to capture correlations or missing predictors (e.g. [28]). Since the among-species correlation matrix **u** has *N*×(*N*-1)/2 entries (*N* being the number of species), which all need to be estimated, a trick is to represent the entries of **u** as a linear function of two or more latent variables, thereby reducing the number of parameters to be estimated (for details see [1]). LVMs are then formulated as mixed-effect models, in their simplest form assuming a Gaussian distribution of abundances, as:

1 $$ m_{\text{ij}} = {\beta}_{{{\text{oj}}}} + {\mathbf{X}}_{{\text{i}}} {\beta}_{{\text{j}}} + {\mathbf{u}}_{{{\text{ij}}}} + \varepsilon_{{\text{i}}} $$

where *m*_ij_ is the abundance of species *j* (*j* = 1, …, *m*) at location *i* (*i* = 1, …, *n*), *ß*_oj_ is an intercept, **X**_i_*ß*_j_ represents the regression coefficients (*ß*_j_) of environmental variables (**X**_i_) and ε_i_ are the regression residuals. **u**_ij_ = **z**_i_ λ_j_ and represents variation that can be captured by construction of new explanatory variables composed of latent variables (**z**_i_) and factor loadings (λ_j_), which represent the strength of the relationship between latent variables and observed variables. These latent variables have resemblance to ordination axis [1, 12] and are treated as random factors to acknowledge they are unobserved.

### Analysis

Following Dormann et al*.* [29], we only considered environmental variables with an |r|< 0.7 to avoid collinearity, choosing the overall least correlated variables for further analysis. Then we used the Watanabe-Akaike information criterion (WAIC; [30]) to define the most parsimonious model, based on all possible subsets. For seagrass areas this resulted in the inclusion of organic content of sediments (%), sediment grain-size fraction > 500 μm (%), sediment grain-size fraction 125–250 μm (%), sediment median grain size (μm), and distance to shore (m) as a proxy for inundation time. For bare sand habitats this included: distance to shore, sediment median grain-size, sediment grain size fraction < 63 μm (%), sediment grain-size fraction > 500 μm, organic content of sediments, and coverage of shell hash (i.e. broken shell fragments; %). For continuous variables, we included quadratic functions to account for patchy distributions across the study site. All environmental variables were standardised to mean 0 and variance 1 [31].

We used Markov Chain Monte Carlo (MCMC) methods to run the LVMs, using JAGS [32] via the package BORAL [13] in R [33], assuming a negative binomial distribution based on a quantile plot of Dunn-Smyth residuals. Default uninformative priors were used for all parameters in all models. We ran models allowing for two latent variables for 125,000 iterations, using a burn-in of 25,000 iterations. Using more than two latent variables would have been possible, but preliminary model runs suggested that such was not necessary to capture patterns of species co-occurrences. R code for fitting and analysis of LVM are available from [1, 12, 13].

## Supplementary information

**Additional file 1: Table S1** CSV-file containing the species-occurrence information for seagrass habitats, as well as associated values for environmental variables.

**Additional file 2: Table S2** CSV-file containing the species-occurrence information for sandy habitats, as well as associated values for environmental variables.

## Data Availability

The datasets supporting this article have been uploaded as part of the additional files.
